# Sex, scarring, and stress: understanding seasonal costs in a cryptic marine mammal

**DOI:** 10.1093/conphys/cot014

**Published:** 2013-06-29

**Authors:** Elizabeth A. Burgess, Janine L. Brown, Janet M. Lanyon

**Affiliations:** 1 School of Biological Sciences, The University of Queensland, St Lucia, QLD 4072, Australia; 2 Center for Species Survival, Smithsonian Conservation Biology Institute, National Zoological Park, 1500 Remount Road, Front Royal, VA 22630, USA

**Keywords:** Body condition, faecal glucocorticoid, male aggression, scarring, stress, tusk injury

## Abstract

We investigated variation in adrenal activity in the dugong, a vulnerable cryptic marine mammal species, in a population inhabiting the lower thermal limit of their range. We sampled 319 live wild dugongs and examined faecal glucocorticoid (fGC) concentrations in relationship to sex, reproductive state (immature, sexually mature, or pregnant), and season, as well as associations with body condition, male-induced aggressive conspecific interactions, and environmental temperature. Physiological validations with apparently healthy and unhealthy animals indicated that fGC levels reliably reflect adrenal activation in dugongs. Glucocorticoid levels and body condition varied seasonally, with individuals expressing lowest fGC levels and an improving body condition over summer and autumn. Best body condition was observed in winter, which may have enhanced the ability of the animals to cope with thermal and nutritional challenges and helped to sustain the subsequent costs of reproduction (including mating activity in spring). Immature dugongs, especially those recently estranged from their mothers, may be particularly vulnerable to stress during winter, and also at risk of injury from adult conspecifics. During the spring mating period, mature and sexually active males with large erupted tusks had high fGC and poorer body condition compared with all dugongs, except pregnant females, in all other seasons. This finding was consistent with high levels of body scarring on adults of both sexes, resulting from agonistic behaviour by mature males. Competitive reproductive behaviour, inherent in a promiscuous mating system, may have stressful consequences for male dugongs, especially given that strenuous mating activity follows unfavourable thermal and nutritional conditions. Overall, fGC levels appear to be a good proxy for stressors in dugongs associated with season and/or temperature, intra-specific aggression, and pregnancy, but not lactation and/or maternal care. Understanding baseline fGC levels will allow wildlife managers to identify additional and/or unusual stressors that may threaten wild dugong populations.

## Abstract (precis)

This study demonstrates that fecal glucocorticoid levels are a useful measure of diverse stressors in dugongs (a vulnerable cryptic marine mammal), including cold temperature and other seasonal factors, intraspecific aggression/injury, and pregnancy. The intrinsic underlying glucocorticoid patterns of dugongs are different between sexes, across reproductive maturity states, and vary seasonally in response to reproductive patterns and environmental conditions.

## Introduction

Environmental perturbations constitute major selective forces for wildlife populations (Wingfield, 2005). An important requirement for any free-living animal is an adjustment of behaviour and/or physiology in response to a changing environment that has both social and physical components. Individuals must cope with predictable seasonal challenges in climate and resource availability (Nelson *et al.*, 2002), but also with unpredictable events, such as extreme weather, agonistic social interactions, and predators. Modern-day stressors may include anthropogenic disturbance, direct exploitation, pollution, and global climate change (McEwen and Wingfield, 2003; Hofmann and Todgham, 2012). Determining the underlying strategies by which animals cope with changing environments has become an important focus in conservation biology, because stress can alter an animal's behaviour and physiology, reduce resistance to disease, inhibit reproduction, and ultimately affect population performance (see Walker *et al.*, 2005).

Dugongs are globally vulnerable wildlife, which are notoriously difficult to study owing to their cryptic nature. They spend little time at the water surface, are unidentifiable by sight alone (Lanyon *et al.*, 2002; Broderick *et al.*, 2007; McHale *et al.*, 2008), and typically live in turbid water of relatively remote areas (Marsh *et al.*, 2002). Dugong population declines have been attributed to human impacts, including coastal development (Marsh *et al.*, 2002), and significant mortality is associated with natural stressors, including cyclones and coastal flooding (Marsh, 1986; Preen and Marsh, 1995). Such perturbations are of major concern for dugong conservation, especially considering their limited dispersal, long lifespans, protracted reproductive cycles (Heinsohn *et al.*, 2004; Marsh and Kwan, 2008), and presumed slow population recovery times (Marsh *et al.*, 2002). The impacts of disturbances on dugong populations have primarily been assessed through declines in abundance or changes in distribution (e.g. Marsh, 1986; Preen and Marsh, 1995; Heinsohn *et al.*, 2004; Marsh *et al.*, 2004). However, it is imprudent to wait for mortality events and/or population movements before initiating a response to manage a given stressor. The identification of normative baseline patterns of adrenal activity has become increasingly useful for management of wildlife, because this allows identification of sensitivities to unusual disturbance (Schwarzenberger, 2007). Until now, we have had only a superficial understanding of stress physiology in sirenians (Tripp *et al.*, 2010; Lanyon *et al.*, 2012), despite the certainty that some sirenian populations may be particularly prone to stress-induced pathologies (Bossart *et al.*, 2002; Lanyon *et al.*, 2011).

The biology of dugongs suggests that a variety of potential environmental stressors may hamper population stability and/or recovery. Dugongs occupy a unique niche as the only herbivorous, fully marine mammals. As seagrass specialists, dugongs are prone to nutritional challenges, because seagrasses are intrinsically low in energy and nutrients, and seagrass growth is susceptible to seasonal fluctuations and longer-term episodic diebacks (Lanyon, 1991; Preen, 1992; Preen *et al.*, 1995). Large-scale deterioration of seagrass habitat across much of the dugong's range (Marsh *et al.*, 2002) is likely to be causing nutritional stress and making populations even more vulnerable. Unlike other marine mammals, sirenians do not accumulate large stores of blubber as energy reserves or insulation (Domning, 1977) and appear to be thermally restricted to warm waters owing to poor thermoregulatory capacity (Worthy, 2001). A lack of cold tolerance is well documented in the related Florida manatee, with ‘cold stress syndrome’ recognized as a major cause of mortality at higher latitudes (Bossart *et al.*, 2002). Dugongs do not exhibit overt pathological manifestations of cold-related stress; however, it is possible that sub-pathological variations in adrenal activity related to thermal challenges affect health and reproductive success. As endothermic herbivores, dugongs at higher latitudes exhibit some behavioural/energetic offsets to temperature drops, including seasonal omnivory (Preen, 1995) and regional movements (Preen, 1992; Lanyon, 2003). It is also possible that dugongs have evolved physiological strategies to avoid the deleterious effects of predictable environmental stressors.

Breeding or social interactions can also be stressful (Boonstra, 2005). Mating behaviour in dugongs is highly seasonal and competitive (Burgess *et al.*, 2012b), with a single female pursued and then embraced by a ‘mounting group’ of four or five males all jostling vigorously for an opportunity to copulate (Preen, 1989). All mature males, and some aged females, are equipped with a pair of erupted tusks, each of which has a sharp, bevelled labial edge (Marsh, 1980). Extensive tusk rake wounds on the dorsum of both sexes present physical evidence of injurious conflicts. As sexually mature males are most capable of inflicting tusk wounds, it is probable that females receive wounds during copulation attempts and that males are injured during competitive interactions with mature males. The elusive nature of dugongs has meant a paucity of information on social behaviour and its effects on individuals. If mating behaviour is energetically strenuous and possibly stressful, then a physiological response during the mating season is likely.

When an animal perceives a stressor, the higher brain areas initiate a complex array of physiological responses that involve both the sympathetic nervous system and the hypothalamic–pituitary–adrenal axis. As part of the stress response, the adrenal gland releases glucocorticoids (i.e. cortisol and/or corticosterone) into the bloodstream within minutes. These hormones function primarily to regulate metabolism and mobilize energy needed to cope with immediate challenges, by stimulating the release of glucose, fatty acids, and triglycerides from storage sites to exercising muscle and the brain (reviewed by Sapolsky, 1992). Although activation of the stress response is clearly adaptive, it is also inherently costly, because long-term elevation of glucocorticoid stress hormones can suppress reproduction (Moberg, 1991; Romero, 2004), inhibit growth, and reduce immune and digestive function (Sapolsky *et al.*, 2000; Romero, 2004). Consequently, glucocorticoid production has been widely measured in wildlife as a means of assessing the physiological costs of behavioural interactions and resource fluctuations (e.g. Creel *et al.*, 1997; Cavigelli, 1999; Strier *et al.*, 1999; Goymann *et al.*, 2001; Creel, 2005; Hunt *et al.*, 2006), and can be symptomatic of exposure to events that may have detrimental effects on population health and survival (e.g. Romero and Wikelski, 2001; Pride, 2005). The assessment of physiological parameters associated with stress is imperative for an understanding of the health and reproduction of wildlife populations (Boonstra, 2005; Reeder and Kramer, 2005), particularly for a vulnerable species, such as the dugong.

This study investigated temporal variation in glucocorticoid levels of wild dugongs at the cold-water limit of their distribution in eastern Australia, Moreton Bay. We theorized that these dugongs activate adrenocortical responses to adjust energy expenditure in response to pronounced environmental seasonality. By measuring faecal glucocorticoid (fGC) concentrations as an index of adrenocortical activity and physiological stress in dugongs, broad patterns of stress physiology were examined across a wild population. Specifically, our objectives were as follows: (i) to identify an appropriate glucocorticoid assay for dugongs by performing a validation using faecal samples from individuals whose hormone content could be predicted *a priori*; (ii) to describe normative patterns of fGC expression in both sexes and all reproductive states of dugongs and to compare levels across seasons; (iii) to examine the influence of sex, reproductive state, and season on dugong body condition and its relationship with fGC production; (iv) to determine the level of male to conspecific interactions and associated physiological stress on all dugongs, using tusk rake injury as an indirect measure of male aggression; (v) to determine the effects of potential predictor variables (maturity state, season, sea surface temperature, frequency of tusk rake injury, body condition, and sexual activity) on physiological stress in wild dugongs; and (vi) to assess whether fGC is a good proxy for certain known stressors, such as pregnancy, lactation/maternal care, cold stress, and aggression/injury in dugongs.

## Methods

### Study site and sample collection

This study was conducted in Moreton Bay, Australia (latitude 27° 20.09′ to 27° 24.87′ S; longitude 153° 21.26′ to 153° 23.84′ E), a locality with the largest dugong population close to a major city (Brisbane), and at the southern limit of dugong distribution on the east coast of Australia (Preen, 1992; Lanyon, 2003). Wild dugongs were sampled over 5 years and across all months (except April) between July 2005 and June 2010, as part of a long-term mark–recapture programme (Lanyon *et al.*, 2002). Seasons were austral summer (December–February), autumn (March–May), winter (June–August), and spring (September–November). Mean monthly sea surface temperature data (in degrees Celsius) for Moreton Bay over the sampling period were obtained from Integrated Marine Observing System, Australian Oceans Distributed Active Archive Centre (CSIRO, 2012).

Dugongs of both sexes and all ages (except neonatal calves) were captured opportunistically across the entire Eastern Banks region of Moreton Bay using an open-water technique (Lanyon *et al.*, 2006). During restraint at the water surface, each dugong was tagged for identification (Lanyon *et al.*, 2002; Broderick *et al.*, 2007) and sexed (Lanyon *et al.*, 2009). Photographs were taken of the dorsum of each dugong to assess the occurrence of tusk rake marks from conspecifics (see ‘Evaluation of conspecific injurious interactions’ below). All dugongs were checked for tusk eruption, a secondary sexual characteristic (Burgess *et al.*, 2012b). Total body length was measured in a straight line from snout to fluke notch, and girth measures were taken at peduncle, anal, maximum (umbilicus), and axillar positions (Lanyon *et al.*, 2010). To measure faecal glucocorticoid metabolites (fGC), ∼4 g of fresh faeces (uncontaminated by seawater) was collected from each dugong by inserting a soft latex tube into the distal rectum. Faecal samples were held on ice before being frozen at −20°C. Health state was assessed visually for each dugong, as indicated by skin condition, muscle tone, signs of disease or infection, and demeanour. Those individuals that could be categorized as ‘apparently healthy’ or ‘apparently unhealthy’ were used in the physiological validation of the glucocorticoid enzyme immunoassays (see ‘Physiological validation of faecal glucocorticoid assay’ below). Dugongs were released within 5–6 min of capture (Lanyon *et al.*, 2006).

### Assigning reproductive state

Sex steroid hormone concentrations of dugong faecal samples, along with body morphometrics, are reflective of the reproductive maturity of dugongs (Burgess *et al.*, 2012a, b). In Moreton Bay, female dugongs with body lengths >260 cm were classed as reproductively mature, while those smaller than 250 cm were never parous and thus probably immature (Burgess *et al.*, 2012a). Pregnancy was diagnosed in female dugongs using faecal progesterone concentration (>1000 ng/g) in combination with body morphometrics, following Burgess *et al.* (2012a). Considering the seasonal breeding activity of dugongs in Moreton Bay (Burgess *et al.*, 2012b), females diagnosed as pregnant within a given season were likely to be at a similar stage of pregnancy, with most births occurring in spring–summer (after 14 months gestation) in this population (Burgess *et al.*, 2012a). Categories of female reproductive states were pregnant, non-pregnant mature (≥260 cm and not associated with a calf), maternal female (confirmed non-pregnant, showing enlarged teats and sighted with a dependent calf), and immature (confirmed non-pregnant and ≤250 cm). Categories of male reproductive states were mature and immature. Male dugongs were classified as mature if their body lengths were >260 cm, while immature males had body lengths <240 cm, low faecal testosterone during the mating season, and unerupted tusks (Burgess *et al.*, 2012b). To achieve a clear distinction between functionally mature and immature individuals, dugongs within the intermediate (pubertal) size range of both females and males were not included in analyses. For cow–calf pairs, the adult member was confirmed as ‘female’ and the dependent calf was confirmed as ‘related’ through genetic analysis (Broderick *et al.*, 2007; McHale *et al.*, 2008). Only large calves (>1 year old) were sampled in this study due to permit regulations.

### Body condition

**Figure 1:**
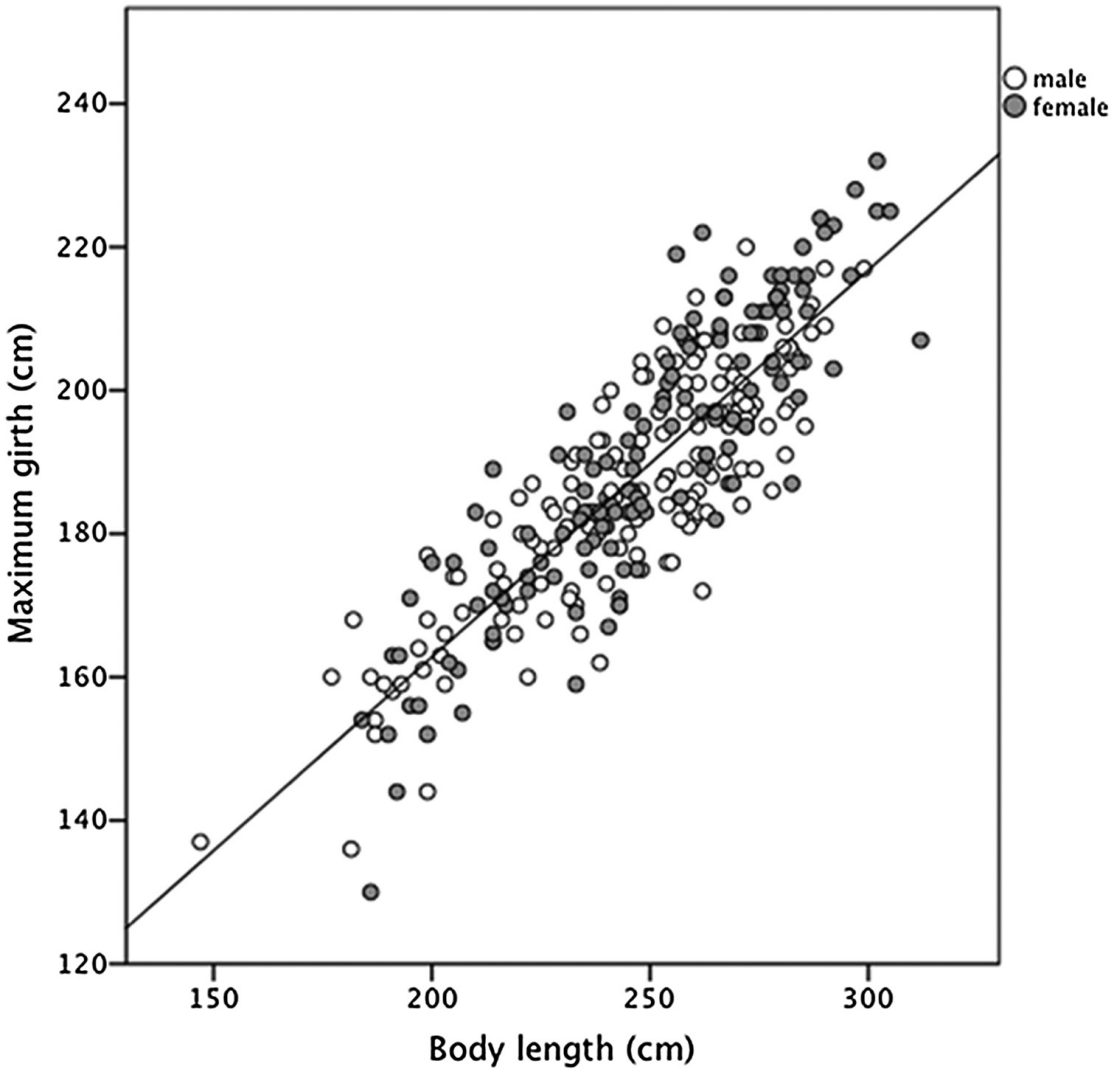
Plot of maximal girth (in centimetres) against body length measurements (in centimetres) for wild dugongs in Moreton Bay (*n* = 291, excluding confirmed pregnant females). Line represents least-squares linear regression fit to the data set, *y* = 55 + 0.5*x* (*r*^2^ = 0.74, *P* < 0.001).

An index of body condition was calculated using a least-squares technique to generate a linear regression of maximal girth against body length, incorporating data from all dugongs (Fig. 1) except pregnant females, because body morphometrics are influenced by gestational stage. Standardized residuals were derived from the regression equation and represented the deviation of each data point (i.e. an individual dugong) from the regression line. These residuals were used as a size-free measure of body condition, with each dugong being assigned a relative body condition measure depending on whether their maximal girth was higher (i.e. positive score = fatter condition) or lower (i.e. negative score = poorer condition) than the population mean (Fig. 1). Similar analytical techniques have been used successfully to remove variation due to size in other morphometric studies (Reist, 1985) and to evaluate body condition indices for marine mammals (Read, 1990; Perryman and Lynn, 2002). Residual values for pregnant female dugongs were independently calculated using the regression equation derived from the sample population of all other dugongs.

### Evaluation of conspecific injurious interactions

The level of aggressive physical interactions between dugongs was gauged by the incidence of dorsal tusk rake mark injury. The act of wounding is rarely observed in dugongs, but tusk-inflicted injuries appear to persist for weeks or months. Tusk rake marks are most abundant on the dorsum of dugongs, and dimensions of these injuries match the distance between the sharp labial edges of the tusks (∼5–6 cm). Tusks erupt at puberty in all male dugongs, and in only a few aged females (Marsh, 1980; Burgess *et al.*, 2012b). Consequently, from the scar patterns on a dugong's dorsum, we inferred the level of aggressive physical contacts received by that individual from a tusked dugong, most probably a reproductively mature male.

**Figure 2:**
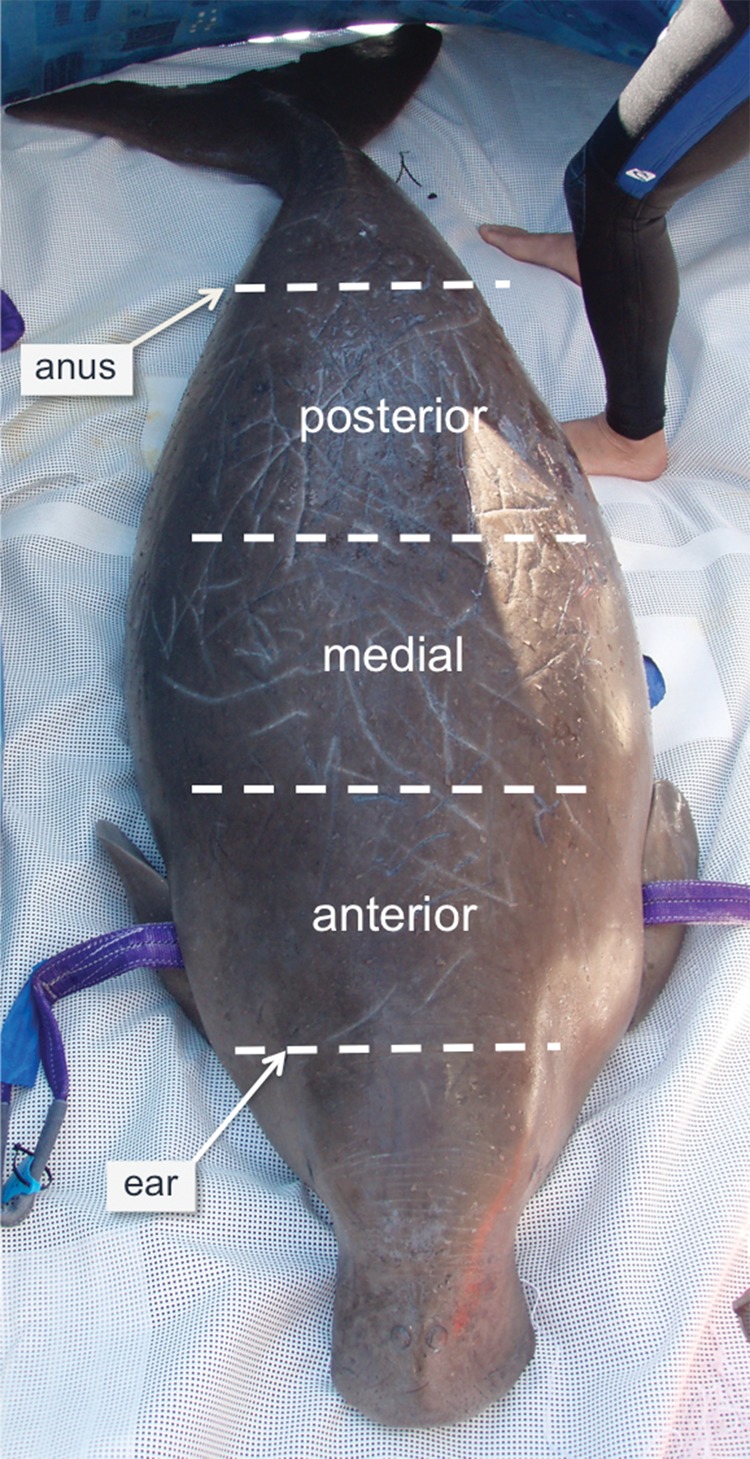
Live dugong, sampled during out-of-water health assessment, showing the three dorsal regions divided for counts of tusk rake injuries: anterior (auditory meatus or ‘ear’ to position caudal of pectoral flipper), medial (pectoral flipper to umbilicus), and posterior (position of umbilicus to anus). Dorsal regions had to be mostly (>75%) or entirely visible in photographs to be included in the analysis.

Photographs were taken of the dorsal surface of each dugong using an Olympus (model 720SW) camera in an underwater housing with a 78 mm wide-angled lens (INON UWL-105-AD). To standardize body surface area, the dorsum was divided into three evenly sized regions: anterior (auditory meatus to caudal insertion of pectoral flipper), medial (caudal insertion of pectoral flipper to umbilicus), and posterior (umbilicus to anus; Fig. 2). To be included in the analysis, photographs had to have >75% of the dorsal region visible and to be of high quality (i.e. good focus, exposure, and contrast).

**Figure 3:**
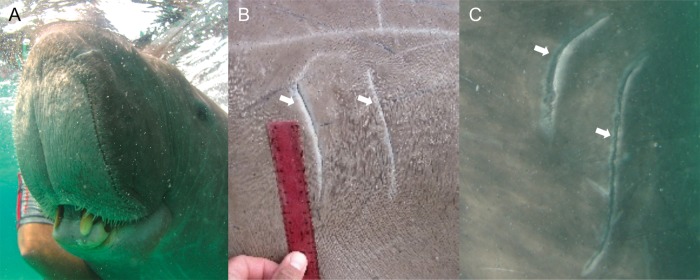
Dugong tusks are a pair of erupted incisors, each of which has a sharp, bevelled labial edge. These erupted tusks are found on all mature males (**A**) and some aged females. Examples of external tusk rake mark injuries caused by paired erupted tusks of another dugong, as shown in magnified photographs of dorsal regions taken out of the water (**B**) and in the water (**C**). Fresh or relatively recent lacerations that had left prominent scars were counted (as indicated by arrows), whilst well-healed or faint rake marks were not included.

Fresh tusk rake marks (Fig. 3) on each dorsal region were counted by magnifying the digital on-screen image and manually tracing each rake mark using *Paintbrush 2.1.1* for Macintosh. Fresh or relatively recent lacerations that had left prominent scars were counted, whereas well-healed or faint rake marks were not included. The total count of rake marks was halved, because it was assumed that the majority of contacts would produce a pair of wounds. This conservative approach may have underestimated the number of injurious interactions if some injuries consisted of single, rather than paired marks.

### Physiological validation of faecal glucocorticoid assay

Faecal glucocorticoid assays always require careful validation, because circulating glucocorticoids (GC) are intensively metabolized in the mammalian gut, yielding a variety of metabolites with unpredictable antibody affinities (Wasser *et al.*, 2000; Palme, 2005; Sheriff *et al.*, 2011). Most standard validations involve conducting adrenocorticotrophic hormone challenges on captive animals to assess adrenal responsiveness (Palme, 2005; Sheriff *et al.*, 2011). However, few dugongs are kept in captivity around the world, and these are shielded from most scientific intervention. Thus, an adrenocorticotrophic hormone experiment was not possible on captive individuals and was considered too invasive on wild dugongs. In order to achieve this objective, we compared fGC of dugongs we suspected, *a priori*, to have variable adrenal activity, similar to physiological validation methods used in studies of large whales (Hunt *et al.*, 2006) and the Florida manatee (*Trichechus manatus latirostris*; Tripp *et al.*, 2010). Health problems are known to initiate a physiological response, particularly glucocorticoid output in mammals (reviewed by Touma and Palme, 2005). Consequently, we compared fGC levels in dugongs visually assessed as ‘apparently healthy’ and ‘apparently unhealthy’. Fourteen dugongs (six males and eight females) were categorized as ‘apparently healthy’, i.e. with no external signs of emaciation, ill health, or injury, and with rotund bodies, firm body muscle tone, clear skin, an active demeanour, and normal behavioural responses. Twelve dugongs (six males and six females) were categorized as ‘apparently unhealthy’, with two or more of the following signs: evidence of infection, disease, or serious injury (*n* = 6); scapulae and vertebral column clearly protruding (emaciation; *n* = 6); abnormal skin (*n* = 8); and/or lethargic behaviour prior to capture (*n* = 6).

We compared the following two antibodies for their capacity to detect the major excreted glucocorticoid metabolites in faeces of apparently healthy and unhealthy dugongs: (i) Cs6 raised against corticosterone; and (ii) R4866 raised against cortisol (both antibodies provided by C. Munro, UC Davis, CA, USA). Glucocorticoid concentrations (log_10_ transformed, expressed as nanograms per gram) of apparently healthy and unhealthy dugongs were compared using Student's paired *t*-test analysis for each antibody EIA. Results showed that Cs6 and R4866 antibodies were both able to detect quantifiable amounts of faecal corticoid metabolites; however, the assays varied in their ability to distinguish between dugong groups. R4866 antibody significantly discriminated fGC measures between health states (paired *t*-test, *t*_24_ = 2.98, *P* = 0.006); apparently unhealthy dugongs had significantly higher fGC concentrations (45.0 ± 2.7 ng/g) than healthy individuals (35.4 ± 2.0 ng/g). In comparison, Cs6 antibody EIA indicated a similar trend, with apparently unhealthy dugongs having higher concentrations of fGC (74.0 ± 11.4 ng/g) than apparently healthy dugongs (52.3 ± 4.6 ng/g), but results did not reach statistical significance (paired *t*-test, *t*_23_ * = * 1.92, *P* = 0.07). This suggests that R4866 antibody EIA may be most appropriate for dugongs.

### Measurement of faecal glucocorticoid metabolites

Faecal steroids were extracted using published methods (Burgess *et al.*, 2012a, b). Samples were oven-dried at 55°C overnight, pulverized, and 0.20 ± 0.01 g of the resulting powder was mixed with 4.0 ml of 80% methanol in a glass vial. Capped vials were vortexed briefly, placed on a rotator overnight (minimum of 12 h), and then centrifuged for 15 min at 3000 r.p.m. (1790*g*). Of the resulting supernatant, 500 μl was aliquoted into a clean glass tube, evaporated to dryness under compressed air, and then reconstituted in 500 μl of standard assay buffer (0.2 m NaH_2_PO_4_, 0.2 m Na_2_HPO_4_, 0.15 m NaCl, and 0.1% albumin bovine serum, pH 7.0). Samples were stored frozen at −20°C until hormone analysis.

Based on the physiological validation tests (see ‘Physiological validation of faecal glucocorticoid assay’ above), dugong faecal extract samples were analysed using the single-antibody R4866 cortisol EIA. This antibody had the following cross-reactivities: 100% cortisol, 10% prednisolone, 6% prednisone, 5% cortisone, and <1% corticosterone, desoxycorticosterone, 21-desoxycortisone, testosterone, androstenedione, androsterone, and 11-desoxycortisol (Young *et al.*, 2004). Assay procedures were similar to those previously described by Young *et al.* (2004). In brief, 96-well microtitre plates were coated with 50 μl of cortisol antibody solution (1:15 000 dilution) and incubated overnight at 4°C. Plates were washed to remove unbound antibody then, immediately after being washed, 50 μl of standard (nine standards were used, spanning 3.9–1000 pg/50 μl), control, or diluted faecal extracts (1:2 to 1:4 in standard assay buffer) and 50 μl of horseradish peroxidase–cortisol conjugate were added to each well. After incubating for 2 h at room temperature, plates were washed, and 100 μl of substrate solution was added to each well. Plates were read when the optical density of the maximal binding wells was 1.0, using a single filter at 405 nm in an optical density plate reader (Dynex MRX Revelation, Dynex Technologies, Chantilly, VA, USA). All samples, standards, and controls were assayed in duplicate, with the resulting coefficient of variation between all duplicates required to be <10% for acceptance. Data were expressed as nanograms per gram of dry faecal weight.

To ensure that the extract medium did not interfere with functioning of the assay, we conducted the following biochemical validations: (i) demonstrated parallelism between serially diluted extracts and the standard curve (*r*^2^ = 0.98, *P* < 0.001); (ii) significant recovery of tritiated cortisol (83 ± 2%) added to dry faecal material before extraction and analysis (extraction efficiency); and (iii) significant recovery of exogenous cortisol (31.25–1000 ng/well) added to faecal extracts before extraction (*r* = 1.005*x* + 0.64). To monitor precision and reproducibility in our assays, high- (30% binding) and low-quality (70% binding) control samples were run on each plate (*n* = 15 assays). Inter-assay coefficients of variation were 6.7 ± 0.6% (high control) and 9.8 ± 3.3% (low control), and intra-assay coefficients of variation were <10%.

Concentrations of sex steroids (androgen and progestagen) in faecal samples from the same dugongs sampled in this study have been presented elsewhere (Burgess *et al.*, 2012a, b) and are used here to interpret glucocorticoid results.

### Statistical analysis

Hormone concentrations were expressed as nanograms per gram ± SEM. Dugongs identified as ‘apparently unhealthy’ for the purposes of assay validation were included in all analyses, because these body conditions are not unusual for dugongs and are important to an understanding of normative patterns. To compare counts of injurious interactions between each dorsum region of dugongs, we used a repeated measure generalized linear model (GLM) with Poisson probability distribution and log-link function. All dugongs included in this analysis had all three dorsal regions photographed, allowing comparison of the frequency of tusk rakes between regions to ensure representative sampling. These results determined whether there was significant bias in tusk rake wounds between regions and helped to identify the dorsal region most appropriate for the examination of a larger proportion of the sampled population. A GLM fitted by maximal likelihood was used to analyse data on body condition (normal probability distribution and identity link) and tusk rake injury counts (Poisson probability distribution and log-link). To analyse fGC data, we used maximal likelihood to fit the GLM with normal errors and an identity link. A full factorial model was used to examine the effects of potential predictor variables (maturity state, season, sea surface temperature, tusk rake injury count, and index of body condition) on fGC concentrations (response variable) in all dugongs, except pregnant females (because fGC is likely to be influenced by gestational stage; e.g. Foley *et al.*, 2001; see Results). Assumptions were tested by visually checking residual distributions and Q–Q plots. The analyses we report use unweighted means. No reported inferences changed when more complex mixed models were fitted. *Post hoc* Bonferroni tests of pairwise multiple comparisons were used to identify all significant differences. To investigate maternal influences further, we compared data (body condition and fGC concentration) of females with associated calves and adult non-pregnant females using Student's independent *t*-test. Likewise, we compared data on small calves (<190 cm) still associated with their mothers with data on unaccompanied dugongs of similar size. Data on fGC concentrations were log_10_ transformed to meet assumptions of normality. A linear correlation analysis was performed to examine the relationship between fGC and body length in small dugongs. All statistical tests were conducted using SPSS^®^ statistical software (version 20.0 for Macintosh; SPSS Inc., Chicago, IL, USA) at a significance level of *P* < 0.05.

## Results

Faecal samples were collected from a total of 319 individual dugongs (157 males and 162 females) and analysed for fGC concentration. Of these samples, 259 individuals (81%) could be definitively assigned to a reproductive state (see Burgess *et al.*, 2012a, b). These included 49 immature males, 61 mature males, 60 immature females, 48 non-pregnant mature females with no associated calf, 28 pregnant females, and three maternal cows, potentially lactating, with dependent calves. We also collected faecal samples from 10 calves (five males and five females) associated with their mothers and potentially nursing, and six (three male and three female) small (<190 cm) calves apparently weaned early from cows (i.e. estranged). Dugongs were sampled year-round across all seasons, except for maternal females (two sampled in summer and one in winter).

### Body condition of dugongs

Mean body condition index was higher (better) in female dugongs (0.13 ± 0.08; excluding confirmed pregnant females) than in males (−0.11 ± 0.09; GLM fitted by maximal likelihood, *b* = 0.13 ± 0.09, Wald statistic = 4.44, *P* = 0.04), with reproductive maturity also significantly influencing body condition (*b* = 0.24 ± 0.12, Wald statistic = 13.47, *P* = 0.004). The lowest scores were measured in mature males (−0.29 ± 0.10), whose body condition was poorer than that of non-pregnant mature females (0.24 ± 0.13; *P* < 0.05). Immature dugongs of both sexes had mean body condition scores (0.00 ± 0.08) intermediate between mature males and non-pregnant mature females (*P* > 0.05). Adult females with dependent calves had poorer body condition (−0.48 ± 0.07) than other adult females (independent *t*-test, *t*_54_ = 3.35, *P* = 0.003). Calves (<190 cm) without an attendant mother had significantly lower body condition (−0.65 ± 0.42) than similar-sized dependent calves (0.37 ± 0.21; independent *t*-test, *t*_16_ = −2.45, *P* = 0.03).

**Figure 4:**
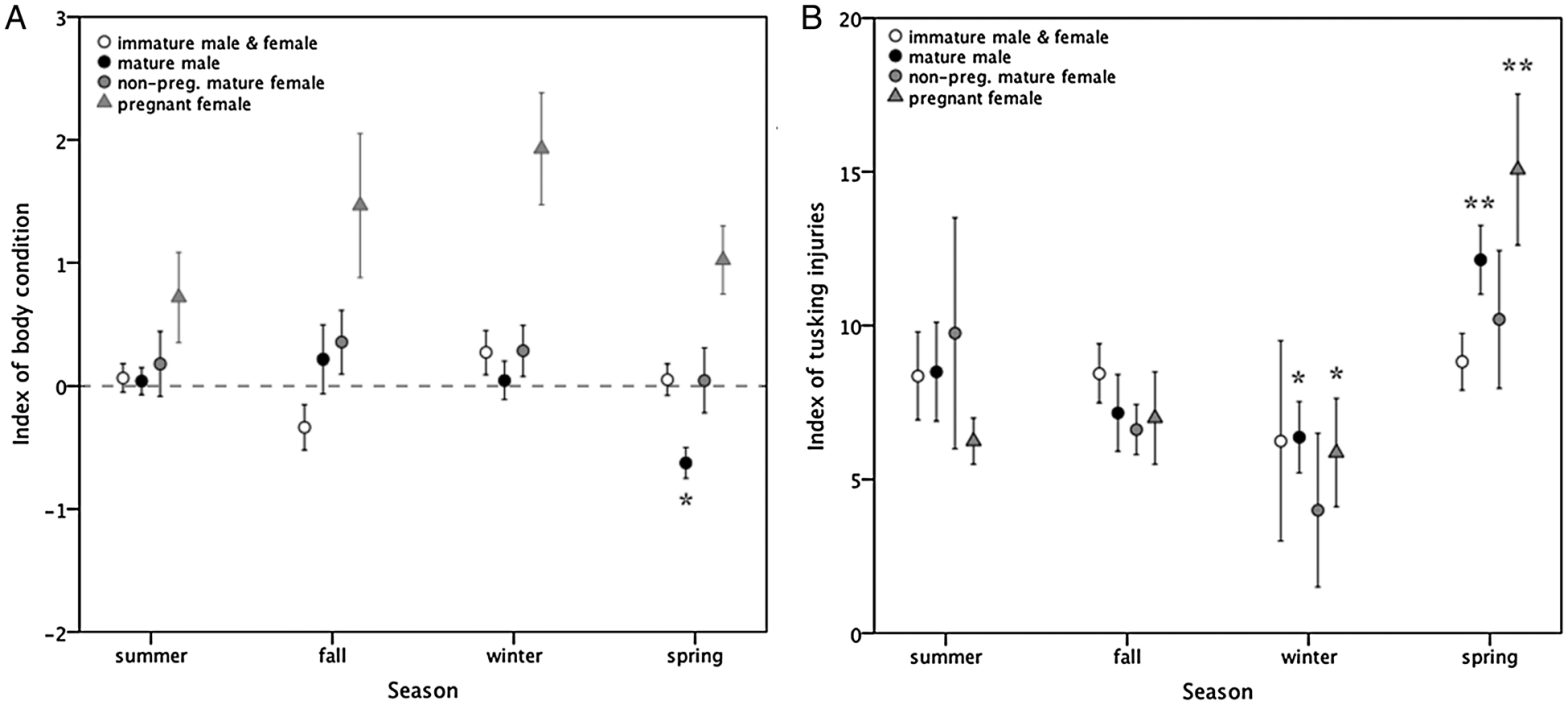
(**A**) Seasonal differences in body condition (standardized regression residuals of body length and maximal girth; note that pregnant females were calculated independently of all other groups and displayed for reference; see Methods). (**B**) Seasonal differences in level of tusk rake injuries on the dorsum. Symbols are as follows: open circles, immature dugongs (both sexes pooled); black circles, mature males; grey circles, non-pregnant mature females; and grey triangles, pregnant females. Values represent means ± SEM. Asterisks denote significantly different groups at *P* < 0.05.

Moreton Bay dugongs (excluding pregnant females) tended towards poorest body condition during spring (−0.11 ± 0.10), with improving condition over summer (0.01 ± 0.08), and best body condition in autumn (0.09 ± 0.12) and winter (0.09 ± 0.11; Fig. 4A). These seasonal trends in dugong body condition varied with reproductive maturity (*b* = 0.13 ± 0.09, Wald statistic = 14.67, *P* = 0.04). In immature dugongs of both sexes as well as in non-pregnant females, body condition was largely unchanged throughout the year (*P* > 0.05), although body condition trended towards best scores during the middle of the year (i.e. autumn and winter cf*.* summer and spring; Fig. 4A). In contrast, the body condition of mature male dugongs was highly influenced by season (*P* < 0.05), with poorest condition during spring (−0.62 ± 0.10), improving over summer (0.04 ± 0.11), reaching a peak in autumn (0.22 ± 0.28), and declining in winter (0.05 ± 0.15; Fig. 4A). In comparison, pregnant females showed increasing body girth in spring (1.02 ± 1.14) and summer (0.72 ± 0.37) through autumn (1.47 ± 0.59), with best condition over winter (1.93 ± 0.45), i.e. during later gestation (Fig. 4A), although differences between seasons were not significant (*P* > 0.05).

### Tusk rake injury on dugongs

The number of injurious encounters, as evidenced by counts of tusk rake marks, differed significantly between the three dorsal regions (anterior, medial, and posterior) of dugongs (Poisson regression GLM repeated measures fitted by maximal likelihood, *b* = 0.84 ± 0.36, Wald statistic = 25.95, *P* < 0.001). The medial region of the dorsum consistently had the highest incidence of rake marks in both sexes (females, 10.8 ± 1.0 tusk marks; and males, 8.7 ± 0.6 tusk marks), with intermediate counts observed on the posterior (females, 8.8 ± 0.8 tusk marks; and males, 8.2 ± 0.7 tusk marks), and lowest counts on the anterior region (females, 8.5 ± 0.9 tusk marks; and males, 6.4 ± 0.6 tusk marks; *P* < 0.05). Consequently, only dugongs with photographs taken of their medial dorsum were used in the whole study.

All 158 dugongs examined (all size groups and sexes) had tusk rake injuries, with between one and 25 (mean 8.8 ± 0.4) recorded on the medial dorsum. Counts of rake marks were significantly higher on reproductively mature dugongs (males, 9.1 ± 0.8; non-pregnant females, 6.5 ± 0.9; and confirmed pregnant females, 10.6 ± 1.2) than immature individuals (males, 8.4 ± 0.6; and females, 9.4 ± 1.2; Poisson regression GLM fitted by maximal likelihood, *b* = 2.36 ± 0.11, Wald statistic = 10.05, *P* = 0.04). However, small (<190 cm) estranged calves had even higher counts of rake marks (12.2 ± 1.4) and twice as many as similar-sized dependent calves (5.7 ± 1.0; *b* = 1.74 ± 0.17, Wald statistic = 13.54, *P* < 0.001). Seasonal trends in counts of rake marks were significantly influenced by reproductive state (*b* = 2.36 ± 0.11, Wald statistic = 10.05, *P* = 0.04). Tusk injuries on immature dugongs of both sexes were consistent across all seasons (Fig. 4B), and a similar non-seasonal trend in tusk injuries was also recorded for non-pregnant mature females (Fig. 4B). In contrast, mature male and confirmed pregnant females had more rake marks in spring (mature males, 12.1 ± 1.1; and pregnant females, 15.1 ± 2.5), less injury in summer (mature males, 8.5 ± 1.6; and pregnant females, 6.3 ± 0.8) and autumn (mature males, 7.2 ± 1.2; and pregnant females, 7.0 ± 1.5), with the lowest injury in winter (mature males, 6.4 ± 1.2; and pregnant females, 5.9 ± 1.8; Fig. 4B; *P* < 0.05).

### Faecal glucocorticoid concentration in dugongs

**Figure 5:**
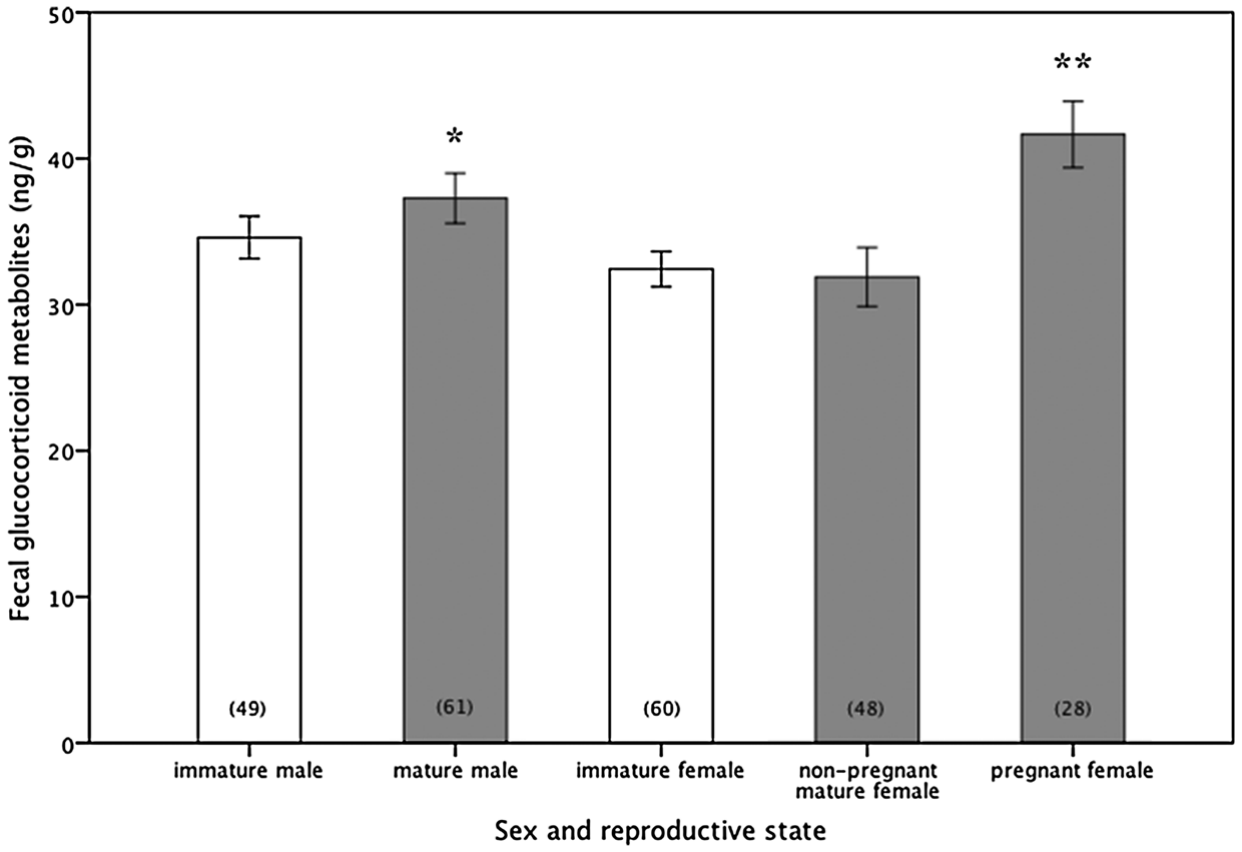
Concentration of faecal glucocorticoid metabolites (expressed as nanograms per gram of dry faeces) in wild dugongs of various sex and reproductive states (open bars, immature group; and grey bars, mature groups). Values are means ± SEM, with samples sizes indicated in parentheses. Asterisks denote significantly different groups at *P* < 0.05.

Faecal glucocorticoid concentrations in dugongs were significantly influenced by sex (GLM fitted by maximal likelihood, *b* = 6.03 ± 2.22, Wald statistic = 16.24, *P* = 0.009) and reproductive maturity (*b* = 41.65 ± 2.20, Wald statistic = 16.24, *P* < 0.001). Male dugongs had significantly higher fGC concentrations (35.9 ± 0.9 ng/g) than females (33.3 ± 0.9 ng/g; *P* < 0.05; all size and maturity cohorts pooled). Pregnant females had the highest fGC (41.7 ± 2.3 ng/g), followed by sexually mature males (37.5 ± 1.8 ng/g), with levels being higher in these groups compared with all others (*P* < 0.05; Fig. 5). Dugongs with the lowest fGC concentrations were immature individuals of both sexes (females, 32.4 ± 1.2 ng/g; and males, 34.6 ± 1.5 ng/g) and non-pregnant mature females (31.4 ± 2 ng/g; *P* > 0.05). Females with dependent calves had fGC levels (27.6 ± 1.1 ng/g) similar to non-pregnant mature females (independent *t*-test, *t*_49_ = −0.3, *P* = 0.76). Dugong calves (<190 cm body length) without an attendant mother had significantly higher fGC (51.4 ± 8.2 ng/g) than calves still associated with cows (30.7 ± 2.6 ng/g; independent *t*-test, *t*_12_ = 3.22, *P* = 0.007). However, the body size of dependent calves was negatively associated with fGC (correlation, *r* = −0.65, *P* = 0.04), with larger calves having lower fGC. This trend was also found among solitary calves, with fGC levels declining significantly with increasing body size (up to 220 cm; correlation, *r* = −0.55, *P* = 0.002), and presumably age.

**Figure 6:**
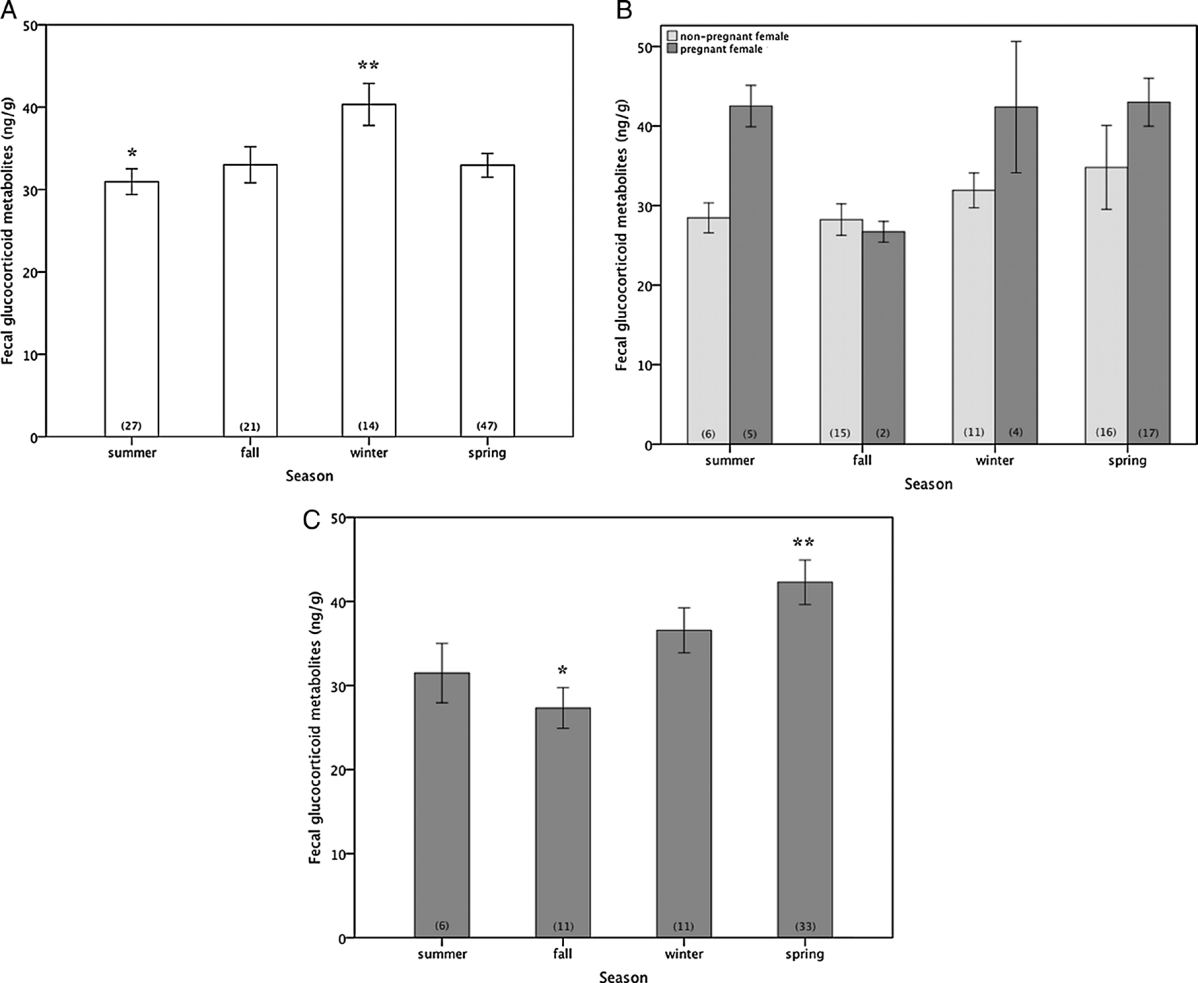
Seasonal variation in faecal glucocorticoid metabolites (expressed as nanograms per gram of dry faeces) of wild dugongs among immature dugongs of both sexes pooled (*n* = 109; **A**), mature female dugongs (**B**), including non-pregnant females (*n* = 48, light bars) and pregnant females (*n* = 28, dark bars), and mature males (*n* = 61; **C**). Values are means ± SEM with sample sizes indicated in parentheses. Asterisks denote seasons that are significantly different from other seasons within reproductive groups at *P* < 0.05.

In Moreton Bay, mean monthly sea surface temperatures were significantly lower in winter (18.1 ± 0.1°C) than summer (27 ± 0.2°C), with intermediate temperatures in autumn (23.4 ± 0.3°C) and spring (23 ± 0.2°C; ANOVA, *F*_3,302_ = 209.41, *P* < 0.001). For all dugongs, fGC varied with season (GLM fitted by maximal likelihood, *b* = 23.38 ± 3.86, Wald statistic = 8.35, *P* = 0.02), with higher levels recorded over winter (36.7 ± 1.3 ng/g) and spring (36.7 ± 1.1 ng/g) compared with other seasons (summer, 32 ± 1.1 ng/g; and autumn, 29.7 ± 1.1 ng/g; *P* < 0.05). However, seasonal patterns of fGC expression differed between reproductive states (*b* = 23.38 ± 3.86, Wald statistic = 15.57, *P* = 0.02). In immature dugongs of both sexes, fGC concentrations were higher in winter (40.3 ± 2.5 ng/g) compared with summer (30.9 ± 1.6 ng/g), with intermediate levels in autumn (33 ± 2.2 ng/g) and spring (32.9 ± 1.5 ng/g; *P* < 0.05; Fig. 6A). Although faecal glucocorticoid concentrations in both non-pregnant and pregnant females were not significantly different across seasons (*P* > 0.05), highest fGC for both reproductive states was recorded in spring (non-pregnant mature females, 34.8 ± 5.3 ng/g; and pregnant females, 43 ± 12.4 ng/g; Fig. 6B). Mature male dugongs had higher fGC over winter (36.6 ± 2.7 ng/g) and spring (42.2 ± 2.6 ng/g) than during other seasons (summer, 31.5 ± 3.5 ng/g; and autumn, 27.3 ± 2.4 ng/g; *P* < 0.05; Fig. 6C). Seasonal changes in fGC concentrations of dugongs in Moreton Bay were strongly influenced by sea surface temperatures (*b* = 29.61 ± 11.6, Wald statistic = 34.35, *P* < 0.001), levels of tusk rake injuries from aggressive conspecific interactions (*b* = 49.68 ± 5.9, Wald statistic = 90.42, *P* < 0.001), and reproductive maturity (*b* = 23.38 ± 3.86, Wald statistic = 41.29, *P* < 0.001), but not by an individual's body condition (*b* = 30.46 ± 1.1, Wald statistic = 3.60, *P* = 0.06).

## Discussion

Adrenocortical activity was reliably reflected in fGC metabolite concentrations of individual dugongs, as shown by biological validation against known health states. Dugongs in noticeably unhealthy condition had significantly elevated fGC levels relative to apparently healthy animals, confirming a stress-axis response similar to those functioning in critically injured wild elephants (Ganswindt *et al.*, 2010), fatally entangled whales (Hunt *et al.*, 2006), and diseased or severely injured Florida manatees (Tripp *et al.*, 2010). Unlike most non-invasive studies, faecal sample collection from wild dugongs in this study required each animal to be captured and restrained. Handling of animals and blood collection may in itself induce glucocorticoid secretion, thereby interfering with the adrenocortical response under investigation (Tripp *et al.*, 2010; Lanyon *et al.*, 2012). However, the approach in this study was unlikely to affect measures of chronic stress, because faeces are less affected by the instantaneous secretion of glucocorticoids than blood, and offer the advantage of evaluating adrenal activity over a period of time related to digesta retention time (Harper and Austad, 2000), i.e. 6–7 days in the dugong (Lanyon and Marsh, 1995). Furthermore, capture-induced acute stress was unlikely to compromise an accurate assessment of adrenal function in this study because the handling period was so short (5–6 min; Lanyon *et al.*, 2006, 2012).

This study established baseline fGC concentrations in wild dugongs of both sexes and various reproductive stages over a period of several years, within a population experiencing natural pronounced seasonal fluctuations in abiotic factors, and in the absence of extreme or unusual environmental stressors. Baseline fGC levels varied with demographic groups of dugongs. Pregnant female dugongs had the highest fGC levels amongst all sex and reproductive states, with concentrations on average one-third greater than in non-pregnant mature females. This finding is not unexpected, because adrenal hormones are often elevated in response to endocrine changes during pregnancy (Challis *et al.*, 2000; Breuner and Orchinik, 2002), as reported in African elephants (*Loxodonta africana*; Foley *et al.*, 2001) and North Atlantic right whales (*Eubalaena glacialis*; Hunt *et al.*, 2006). From an energetic perspective, lactation as well as gestation is likely to be expensive metabolically for female mammals (Wade and Schneider, 1992). Such potential costs were evident in maternal dugongs that had poorer body condition, yet similar low glucocorticoid levels to non-pregnant mature females. This finding suggests that lactation and caring for a calf may cause a negative energy balance, but does not affect stress levels as measured by fGC. Resting adult females (i.e. mature but not pregnant, lactating, or accompanied by a dependent calf) did not suffer seasonal declines in body condition, and this maintenance of condition (or at least no significant loss) may allow energetic preparation for future pregnancy.

High levels of fGC were also found in estranged calves that would normally accompany their mothers at this size (i.e. <190 cm; (JM Lanyon, unpublished data). Most of these solitary calves had poor body condition and a high incidence of tusk rake wounds compared with nursing calves, suggesting that they may have been both nutritionally compromised and exposed to more aggressive encounters from mature males. A normal mother–calf relationship in Moreton Bay appears to be protracted, so that the calf is fed and protected over many years until it reaches a body length of around 225 cm (JM Lanyon, unpubliblished data). Two of the six estranged calves sampled in this study were recovered as carcasses the following winter, suggesting that their high fGC levels reflected the early stages of a serious decline in health. In contrast, attendant calves had low fGC levels, generally excellent body condition (always fatter than the cows), significantly lower incidence of tusk rake wounds, and presumably a higher survival rate. The underlying high fGC levels of young estranged or recently weaned dugongs suggests that this cohort may be amongst the most vulnerable when exposed to external environmental stressors or even under unfavourable seasonal conditions.

Seasonal changes in adrenal function and body condition were pronounced among wild dugongs in subtropical Moreton Bay. Over winter, dugongs of all age classes exhibited increasing adrenal activity, which may reflect a physiological adaptation to survival under unfavourable seasonal conditions at this high latitude. In Moreton Bay, winter water temperatures drop as low as 15–19°C, and if dugongs are to maintain body temperatures between 27 and 30°C (Lanyon *et al.*, 2010), elevations in circulating glucocorticoids may assist maintenance of thermal homeostasis. In addition to physiological mechanisms, dugongs in Moreton Bay undertake small-scale movements into warmer oceanic waters in winter (Preen, 1992; Lanyon, 2003), with such behavioural changes presumably helping to offset metabolic insufficiencies. Winter declines in ambient temperature coincide with a seasonal reduction in nutrient availability for dugongs in Moreton Bay (Lanyon *et al.*, 1989; Lanyon, 1991; Nichols, 2005), with increases in glucocorticoids associated with reduced caloric intake possibly indicating a shift to catabolic metabolism. Climatic factors and food availability have both been reported to affect glucocorticoid output in a variety of species (Muller and Wrangham, 2004; Weingrill *et al.*, 2004), and this response is generally most pronounced during cold seasons in response to energy demand (St Aubin *et al.*, 1996; Bubenik *et al.*, 1998). Furthermore, dugongs generally had poorer body condition in the period following winter seagrass dieback, with improved condition during summer and autumn months following periods of seagrass growth. Similar reductions in body condition with concomitant elevations in glucocorticoid levels were also reported for wild African elephants during unfavourable dry seasons, when rainfall and food availability declined (Foley *et al.*, 2001). In strongly seasonal environments, mammals usually accumulate and subsequently lose energy stores in an annual body mass gain–loss cycle (Kurita *et al.*, 2002; Pusey *et al.*, 2005). At least part of the decline in body condition coincident with higher glucocorticoid production in dugongs may be due to depletions of fat reserves during winter. Results of this study suggest that investigations into thyroid hormones (i.e. thyroxine and triiodothyronine, also measurable in faecal samples; Wasser *et al.*, 2010) may yield further insight into nutritional and metabolic processes in wild dugongs.

Elevated fGC levels in winter were most pronounced in immature dugongs (cf*.* mature dugongs with larger body sizes), possibly because these actively growing individuals have metabolic requirements up to twice those of adult sirenians (after Worthy, 2001). However, these young dugongs did not lose body condition throughout the year, and it is likely that excess energy reserves were directed towards growth rather than accumulating fat. In manatees, susceptibility to cold stress syndrome (Bossart *et al.*, 2002) appears to be related to body size, with a predominance of juvenile to subadult-sized manatees dying of hypothermia during severe winters (O'Shea *et al.*, 1985). Juveniles, in particular, may experience increased physiological adjustments during winter, coupled with the competing demands for physical growth and development, as reported for juvenile Steller sea lions (*Eumetopias jubatus*; Mashburn and Atkinson, 2008). It appears that winter may be a critical period for dugongs of this age class. It is also likely that energy constraints may affect growth rate and time to maturity in young dugongs, particularly in areas with pronounced seasonality.

Mature dugongs of both sexes showed trends towards increasing fGC levels over spring, when increased energetic demands were presumably associated with reproductive competition and mating. However, this seasonal effect was significant only for male adult dugongs (cf. mature females), which is consistent with increased adrenal activity associated with male mating behaviour in other seasonally reproducing terrestrial (reviewed by Romero, 2002) and marine mammals (Mashburn and Atkinson, 2007). Male dugongs also sustained more tusk injury in spring, suggesting that male competition is most intense during this period of heightened testosterone production (Burgess *et al.*, 2012b). In addition to high stress, mature male dugongs suffered significant loss of body condition over spring, with improving condition coincident with summer seagrass blooms (Preen, 1992). Presumably, this loss of body condition in males could result from the energetic expenditure of competitive mating (Preen, 1989), coercive copulation (Athousis, 2012), a focus on mating to the detriment of foraging, and/or increased dispersal during the mating season (Burgess *et al.*, 2012b), following a seasonal decline in seagrass nutrients. A similar loss of body condition was not found in reproductive females, suggesting a difference in the energetic demands and life-history strategies of the sexes. Mating activity and intra-sexual competition appears to involve a relatively high energetic cost for male dugongs, as occurs in many other mammalian males (Boonstra, 2005). As a result, male dugongs with higher energy status could presumably invest more in reproductive activities to maximize their chances of reproductive success.

All dugongs in Moreton Bay have tusk-inflicted injury, suggesting that aggressive encounters are common, with each dugong receiving an average of between eight and nine tusk injuries to the dorsum. These conspecific interactions are a significant source of stress for recipient dugongs, with elevated fGC levels being associated with higher frequencies of injury across all size classes. Increased glucocorticoids in recipients may aid in their defense against a tusked male by providing the energy needed for defense or flight. The risk of injury from conspecifics' tusks increased with attainment of maturity for both sexes. Of all dugongs, pregnant females had the highest number of fresh tusk injuries, with almost twice as many rake marks as non-pregnant females. This finding suggests that injury may have been sustained during copulatory attempts, and that the risk of injury in females may increase during times when they are sexually receptive (i.e. spring). In many polgynous species, females are subjected to harassment, either directly as males try to gain mating access or monopolize females, or indirectly through aggressive interactions between males (Linklater *et al.*, 1999; Cappozzo *et al.*, 2008). Mature but non-pregnant female dugongs had lower levels of tusk injuries, similar to immature dugongs. Among immature dugongs, the prevalence of tusk injury was greatest among small solitary calves, suggesting that young dugongs without maternal protection are more vulnerable to male aggression.

In conclusion, this study demonstrates that fGC levels are a useful measure of diverse stressors in dugongs, including cold temperature and/or other seasonal factors, intra-specific aggression and/or injury, and pregnancy, but not lactation and/or maternal care. Furthermore, the intrinsic underlying fGC patterns of dugongs are different between sexes, across reproductive maturity states, and vary seasonally in response to reproductive patterns and environmental conditions. For dugongs living in strongly seasonal environments, such as in Moreton Bay, the annual cycle of reproduction and cold temperatures requires the stress axis to be modulated for reproduction and/or survival in the face of environmental challenges. Such mechanisms of allostasis (i.e. achieving stability by means of a systematic, co-ordinated response to environmental cues; McEwan and Wingfield, 2003) have presumably evolved to cope with predictable environmental change. However, not all stressors can be predicted. Dugongs in Moreton Bay comprise the largest population that lives in close proximity to a major urban centre (Lanyon, 2003), with increasing anthropogenic pressures. The stress response of dugongs to unpredictable alterations to habitat and external stressors, i.e. boat traffic, habitat disturbance, seagrass loss, and extreme weather events, is likely to be superimposed upon this baseline of fluctuating stress. The impacts of environmental challenges on populations are difficult to quantify in long-lived, slowly reproducing species, such as dugongs, because many events may transpire before the survival and reproductive consequences of the disturbances are manifested at the population level. This study provides important baseline data that can be used as a practical and unbiased tool to evaluate the effects of climatic and/or human-induced impacts on vulnerable wild dugongs.
